# Comprehensive Analysis of Expression and Prognostic Value of GATAs in Lung Cancer

**DOI:** 10.7150/jca.52623

**Published:** 2021-05-05

**Authors:** Chengwu Gong, Yun Fan, Xueliang Zhou, Songqing Lai, Lijun Wang, Jichun Liu

**Affiliations:** 1 Department of Cardiothoracic Surgery, Second Affiliated Hospital, Nanchang University, Nanchang, Jiangxi 330006, China.; 2 Department of Neurology and National Clinical Research Center for Aging and Medicine, Huashan Hospital, Fudan University, Shanghai 200040, China.; 3 Department of Cardiothoracic Surgery, First Affiliated Hospital, Nanchang University, Nanchang, Jiangxi 330006, China.

**Keywords:** GATAs, lung cancer, expression pattern, prognosis, database

## Abstract

GATAs are a family of transcription factors that play sophisticated and extensive roles in cell fate transitions and tissue morphogenesis during embryonic development. Emerging evidence indicate that GATAs are involved in tumorigenesis of lung cancer (LC). However, the distinct roles, diverse expression patterns and prognostic values of six GATA family members in LC have yet to be elucidated. In the present study, the diverse expression patterns, prognostic values, genetic mutations, protein-protein interaction(PPI) networks of GATAs, Gene Ontology enrichment and Kyoto Encyclopedia of Genes and Genomes pathway in LC patients were analyzed using a serious of databases, including ONCOMINE database, Cancer Cell Line Encyclopedia database, the Human Protein Atlas, the Gene Expression Profiling Interactive Analysis database, the Kaplan-Meier plotter, cBioPortal, String database and database Database for Annotation, Visualization, and Integrated Discovery. The mRNA expression levels of GATA1/2/4/5/6 were downregulated, while GATA3 showed abnormal expressions of up-regulation and down-regulation in patients with LC. Aberrant GATAs mRNA expression was connected with prognosis. Furthermore, genetic alterations mainly appeared in GATA4. Gene Ontology enrichment and network analysis demonstrated that GATAs and their 50 interactors were primarily associated with positive regulation of transcription from RNA polymerase II promoter, transcription factor complex, transcription factor binding Jak-STAT signaling pathway. This comprehensive bioinformatic analysis demonstrated that GATA1/2/3/4/6 may be new prognosis factors, and GATA2/5/6 may be potential targets for personalized therapy for patients with LC, but further studies are requisite to analyze the mechanism of their carcinogenicity and investigate novel drug treatment. Finally, these findings would conduce to a better understanding of the unique roles of GATAs in LC.

## Introduction

Lung cancer (LC) ranked first in cancer incidence and mortality, with 2.1 million new cases and 1.8 million cancer deaths predicted in 2018, accounting for approximately one-fifth (18.4%) of all cancer-associated mortalities 1. LC can be divided into small cell lung cancer (SCLC) and non-small cell lung cancer (NSCLC) based on histological type. And the latter can be classified as adenocarcinoma, squamous cell carcinoma and large cell carcinoma, which accounts for about 85% of all cases of LC 2. Although emerging treatments such as targeted therapy and immunotherapy have made significant progress, the 5-year survival rate for LC patients varies from 4-17% due to stage and regional differences 3. Furthermore, direct inhalation of tobacco smoke is the leading cause of most lung cancer worldwide, but about a quarter of lung cancer patients never smoke, and their deaths may be attributed to an unfortunate combination of genetics and environmental factors 4. Therefore, better prognosis and individualized treatment require new prognostic markers as well as potential drug targets.

GATAs are a family of transcription factors that play sophisticated and extensive roles in cell fate transitions and tissue morphogenesis during embryonic development. The naming of GATA factors is based on the fact that the zinc-finger domains shared by all family members can recognize the uniform DNA-binding sequence (A/T)GATA(A/G) 5. Although six GATAs are initially classified as hematopoietic (GATA1/2/3) and cardiac (GATA4/5/6) GATA factors based on tissues, their expression patterns and functions are obviously more widespread. The expression of GATAs is aberrant in several common human carcinomas such as breast cancer 6, gastric cancer 7, leukemia 8, bladder cancer 9, ovarian cancer 10 and LC 11.

Up to now, some GATAs exhibited aberrant expressions and their prognostic values in LC. For example, the expression of GATA2 in human and mouse lung tumors were distinctly downregulated compared with normal lung tissues and its further suppression was not an effective treatment for KRAS mutant lung cancer 12, while GATA2 was essential for survival of KRAS mutant NSCLC cells 13. GATA3 was expressed in PC-9 and QG56 LC cell lines, but not in A549 and 11-18 cell lines. Meanwhile, of 95 lung adenocarcinoma cases, positive cells were found immunohistochemically in at least one field in 70 patients (73.7%) and were able to be evaluated. Moreover, the Kaplan-Meier curves demonstrated that elevated GATA3 expression group exhibited worse overall survival (OS) and disease-free survival (DFS) than the low-expression group 14. And in Halla's study 15, GATA3 expressed in 2% primary lung adenocarcinoma cases and in 20% primary squamous cell lung carcinoma cases. GATA4 expression was consistently reduced in human lung cancer cell lines compared with normal lung epithelial cell lines and decreased GATA4 level in clinical specimens predicted poor prognosis 16. Naoki et al. reported that only 47 of 348 cases of lung adenocarcinoma had positive GATA6 expression (13.5%) and GATA6 has no significant influence on OS or DFS using multivariate analysis 17, while GATA6 was remarkably reduced in squamous cell lung carcinoma tissues and can inhibit the proliferation and migration of squamous cell lung carcinoma cells 11.

Besides, in some studies, the clinical relationship between GATA expression and primary lung cancer was not the main object of study. For example, GATAD2B is a member of the Nucleosome Remodeling complex (NuRD), 1 of 4 major ATP dependent chromatin remodeling complexes. The major roles of the complex are regulation of transcription, chromatin assembly, cell cycle progression, and genomic stability 18. Zhang et al. 19 elaborated that a long non-coding RNA, GATA2-AS1, repressing NSCLC cells proliferation via regulating GATA2. GATA2-AS1 gene is located at antisense strand of GATA2 on chromosome while GATA2-AS1 RNA interacts with GATA1 protein at promoter region of GATA2 and then inhibits its transcription. However, the relationship between GATA2 or GATA1 and the clinical and prognosis of lung cancer has not been clarified. Lindholm 20 reported that expression of GATA-6 transcription factor in metastatic pulmonary adenocarcinoma not in primary lung cancer.

However, they just showed a certain aspect of GATA in a certain lung cancer subtype and the expressions of GATAs were inconsistent. What is more, these results are single-center and not representative. In order to obtain more accurate results, we integrated all available data. In addition, GATAs is intended to be used as a prognostic tool or therapeutic strategy in clinical practice as mentioned in some articles, and more studies are still needed to further verify and systematically integrate multi-center results to draw corresponding conclusions. Therefore, the expression in patients with different pathological types and cell lines of LC, mutation patterns, potential functions and their prognostic values of GATAs in LC were investigated by the comprehensive analysis of some large databases to clarify diverse expression patterns and prognostic values of six GATA family members in LC.

## Materials and Methods

### Criteria for patient selection

We included primary lung cancer patients, including SCLC, squamous cell lung carcinoma, lung adenocarcinoma, large cell lung carcinoma and lung carcinoid tumor. And patients with metastatic lung cancer were excluded. Moreover, detailed criteria for the inclusion and exclusion of patients were displayed in the corresponding methods section below.

### ONCOMINE Database

The ONCOMINE database (www.oncomine.org) is a comprehensive online cancer microarray database of tumor-related gene expression. In this study, transcriptional expressions of six different GATAs between different cancer tissues and their corresponding normal samples were acquired from ONCOMINE database. The search contents and thresholds of our analysis were set as follows: P<0.01; fold-change, 2; gene rank, top 10%; analysis type, cancer vs. normal; and data type, mRNA. The *P* value was calculated using the Student's t test. Moreover, five different pathological types of lung cancer included SCLC, squamous cell lung carcinoma, lung adenocarcinoma, large cell lung carcinoma and lung carcinoid tumor.

### Cancer Cell Line Encyclopedia (CCLE) database

The Cancer Cell Line Encyclopedia (CCLE, https://portals.broadinstitute.org/ccle) is an available online encyclopedia to constitute enormous multifunctional profiling datasets across almost 1,000 cell lines from more than 20 cancer types. It compiled gene expression, DNA copy numbers, massively parallel sequencing, histone profiling, RNA-seq, DNA methylation, microRNA (miRNA) profiling, and whole-genome sequencing, and metabolite profiling 21, 22. The mRNA expression levels of GATAs in a series of cell lines for different kinds of human cancer were integrated using CCLE, including non-small lung cancer, small cell lung cancer and chronic myelocytic leukemia, and marked as 'lung_NSC', 'lung_small_cell' and 'CML', respectively in Figure 2.

### Human Protein Atlas

The Human Protein Atlas (https://www.proteinatlas.org) is a public website that to determine global protein expression patterns by analyzing and extracting the included human specimens and clinical material from cancer patients, including images of immunohistochemistry (IHC) in nearly 20 types tumors 23. In the present study, we compared the protein expression of 5 different GATAs in LC tissues and normal lung tissues by immunohistochemistry image.

### GEPIA Database

The Gene Expression Profiling Interactive Analysis (GEPIA) database (http://gepia.cancer-pku.cn/), a neoteric online tool, provides many important interactive and customizable functions. In this study, GEPIA was used to analyze the mRNA differential expression levels of GATAs between tumor and normal, investigate the expression profiling plotting based on cancer types or different pathological stages, and explore the correlation analysis between GATAs.

### The Kaplan-Meier Plotter

The Kaplan-Meier plotter (http://kmplot.com/analysis) is an open online dataset that detects the association of gene expression with survival of patients of some cancers, including lung cancer. In our study, the Kaplan-Meier plotter was used to disclose the prognostic value of mRNA expression of distinct GATAs in LC patients. Cancer patients were divided into high and low expression groups in accordance with median expression. Information about OS, progression-free survival (FP), post-progression survival (PPS), the hazard ratio (HR) with 95% confidence intervals (CIs) and log-rank P values can be found at the K-M plotters. *P*<0.05 means a statistically significant difference.

### TCGA data and cBioPortal

The lung adenocarcinoma (TCGA, Firehose Legacy) dataset including data from 584 cases with pathology reports and squamous cell lung carcinoma (TCGA, Firehose Legacy) dataset including data from 511 cases with pathology reports were selected for further analyses of GATAs by using cBioPortal (http://www.cbioportal.org). These selected genomic profiles included mutations, putative copy-number alterations (CNA) from GISTIC, mRNA expression z-scores (RNA Seq V2 RSEM) and protein expression Z-scores (RPPA).

### The Gene Expression Omnibus (GEO) database

The microarray datasets GSE6044, GSE19188 and GSE21933 were downloaded from GEO database to get gene expression data. Therefore, to further determine prognostic value of GATAs in patients with LC, we obtained survival probability data from some microarray datasets of GEO database, including GSE157011, GSE63459, GSE116959 and GSE50081. The data acquisition and processing were done through R Version 4.0.4.

### String Database and DAVID

The String Database (https://string-db.org/) is a publicly available online that predicts functional associations between different proteins. In this study, the network for GATAs and the 50 most frequently neighbor genes was constructed using the String Database. Minimum required interaction score was selected as high confidence (0.7). Next, Database for Annotation, Visualization, and Integrated Discovery (DAVID) (https://david.ncifcrf.gov/) was used to perform Gene Ontology (GO) and Kyoto Encyclopedia of Genes and Genomes (KEGG) analyses with 6 GATAs and 50 associated proteins. “Homo sapiens” was selected and *P*<0.05 means a statistically significant difference.

## Results

### Expression levels of GATAs mRNAs and proteins in patients with LC

**Figure 1:**
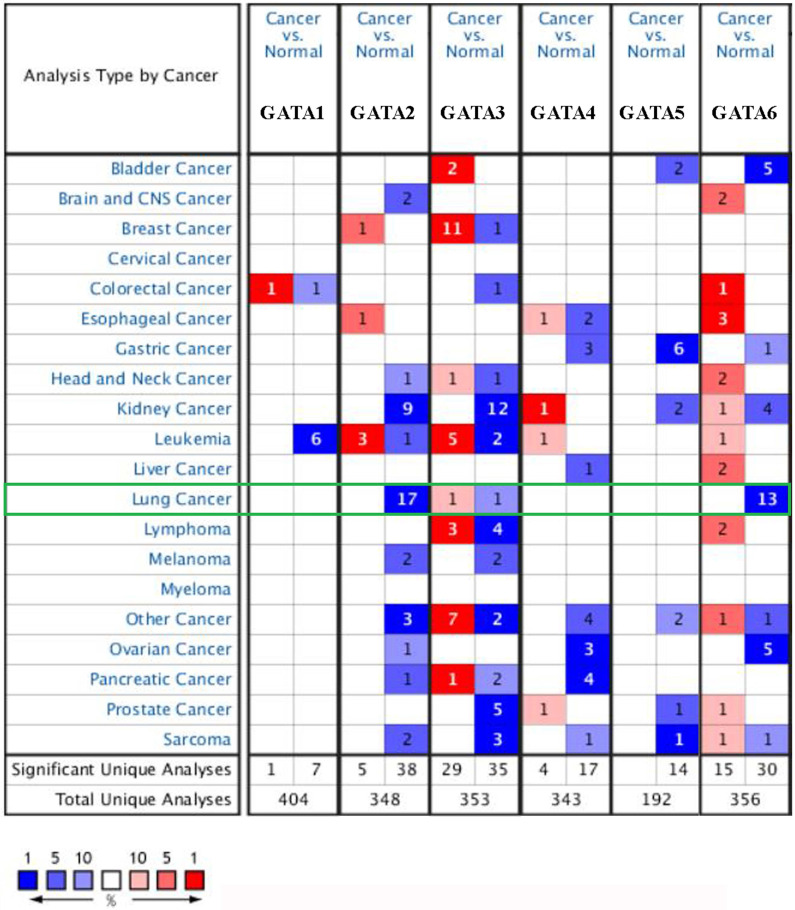
** The mRNA levels of GATAs in different types of cancers (Oncomine).** The number in each cell represents the number of analyses that satisfied the following threshold: *P* < 0.01, the absolute value of log2 fold change >2, and gene rank, top 10%. The numbers in colored cells show the quantities of datasets with statistically significant mRNA overexpression (red) or downexpression (blue) of target genes.

We found the dysregulated transcriptional levels of 6 GATAs in 20 different types of cancers in comparison with normal samples utilizing the ONCOMINE database (**Figure 1**). As shown in Figure 1 and **Table 1**, the mRNA expression levels of GATA2 and GATA6 were markedly downregulated in patients with LC in some datasets while GATA3 had a contradictory level of expression. In Bhattacharjee dataset 24, GATA2 has a lower expression than normal samples in four different LC types: SCLC with a fold change (FC) of -13.297, squamous cell lung carcinoma with a FC of -8.029, lung adenocarcinoma with a FC of -6.027, and lung carcinoid tumor with a FC of -12.984. Furthermore, the results from different datasets showed that there were -4.430-fold, -2.451-fold, -2.552-fold, -2.361-fold, -2.218-fold and -3.332-fold decrease in GATA2 mRNA expression in lung adenocarcinoma, respectively 25-29. And Wachi et al. 30 indicated that the mRNA expression levels of GATA2 were remarkably downregulated in patients with squamous cell lung carcinoma with a FC of -2.142. In Hou's dataset 31 and Garber's dataset 32, the transcription levels of GATA2 in squamous cell lung carcinoma, lung adenocarcinoma and large cell lung carcinoma were both lower than those in lung tissues, and their FC are -3.780/-2.475/-4.077 and -3.551/-2.318/-2.194, respectively. In addition, Beer et al. 28 showed that the mRNA expression levels of GATA3 were prominently downregulated in patients with lung adenocarcinoma with a FC of -2.996 while Garber et al. 32 indicated that GATA3 has a FC of 2.590 in patients with large cell lung carcinoma. Besides, in Wachi's dataset 30 and Bhattacharjee's dataset 24, the decrease of GATA6 was found in squamous cell lung carcinoma compared with normal samples with a FC of -4.710 and -2.600, respectively, while there were -5.083-fold, -3.568-fold, -2.368-fold and -3.392-fold decrease in GATA6 mRNA expression in lung adenocarcinoma, respectively 26-29. Garber et al. 32 showed that GATA6 has a lower expression than normal samples in four different LC types: squamous cell lung carcinoma with a FC of -3.793, large cell lung carcinoma with a FC of -3.039, lung adenocarcinoma with a FC of -3.244, and SCLC with a FC of -5.177. Moreover, in Hou's dataset 31, the transcription levels of GATA6 in large cell lung carcinoma, lung adenocarcinoma and squamous cell lung carcinoma were significantly downregulated, and their FC are -8.949, -4.042 and -6.186, respectively.

In addition, we also verified expression levels of GATAs in patients with LC through some GEO microarray datasets. GATA1 has a lower expression than normal samples in LC types with FC of 1.032 (*p* = 0.014) [GSE6044]. And the mRNA expression levels of GATA1 and GATA2 were markedly downregulated in NSCLC with a FC of 1.148(*p* = 0.003) and 1.123(*p* = 0.006) [GSE19188], while GATA2-5 have different FC of 0.804 (*p* = 8.38E-10), 0.889 (*p* = 0.004), 1.121 (*p* = 0.022), 0.882 (*p* =1.85E-4) and 0.744 (*p* =3.14E-06) [GSE21933], respectively.

**Figure 2:**
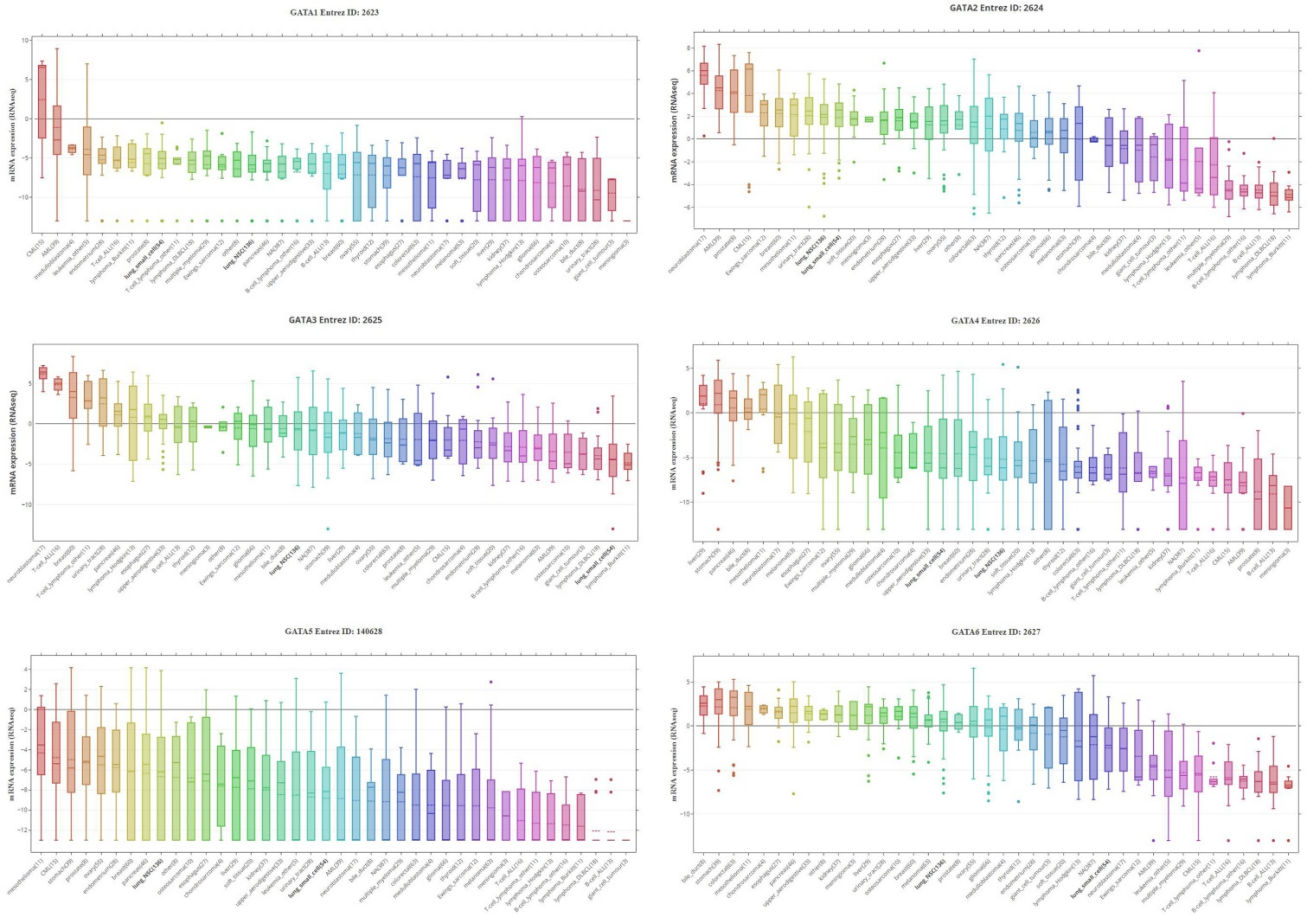
** GATAs protein family expression across 4,103 primary tumors (Cancer Cell Line Encyclopedia database, CCLE).** Box-and-whisker plots showed the distribution of GATA1-6 mRNA expression for each subtype. NSC, non-small cell, DLBCL, diffuse large B-cell lymphoma; CML, chronic myeloid leukemia; AML, acute myeloid leukemia; ALL, acute lymphoblastic leukemia.

By assembling the CCLE, the mRNA expression profiles of GATAs in different kinds of human cancer cell lines were displayed (**Figure 2**). As shown in Figure 2, GATA1, GATA4 and GATA5 were exceptionally downregulated in lung cancer cell lines, including SCLC and NSCLC. On the contrary, levels of GATA2 were upregulated in two lung cancer cell lines. Interestingly, levels of GATA3 and GATA6 were distinctively downregulated in SCLC, while their expressions in NSCLC were not quite explicit.

**Figure 3:**
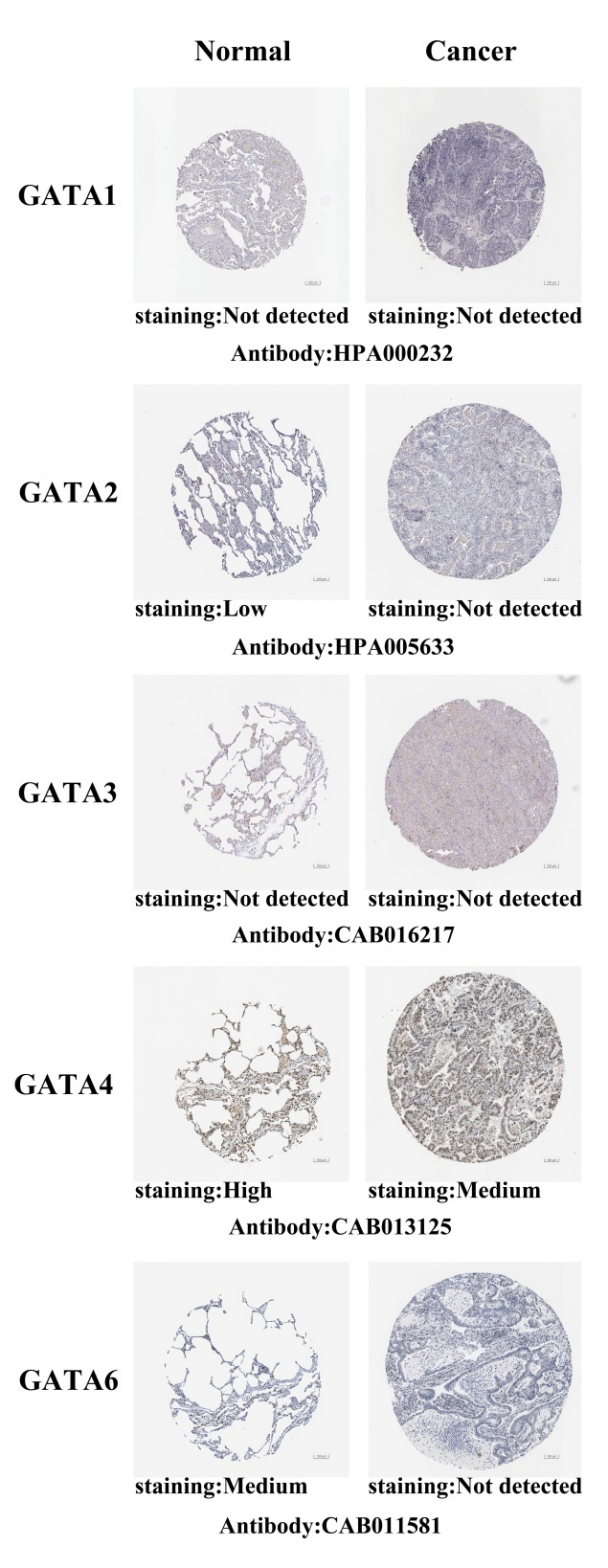
** Representative immunohistochemistry images of distinct GATAs family members in LC tissues and normal lung tissues (Human Protein Atlas, HPA).** GATA1/3 proteins were not expressed both in LC tissues and normal lung tissues. GATA2/6 proteins were not expressed in LC tissues, whereas their low and medium expressions were observed in normal lung tissues. Medium protein expressions of GATA4 were found in LC tissues, while its high protein expressions were observed in normal lung tissues. The protein expression level of GATA5 was not found in HPA.

After exploring the mRNA expression patterns of GATAs in LC, we attempted to find the protein expression profiles of GATAs in LC by the Human Protein Atlas. As shown in **Figure 3**, GATA1/3 proteins were not detected in normal lung tissues and LC tissues. In addition, GATA2/6 also were not detected in LC tissues, whereas low and medium expressions of them were observed in normal lung tissues. High protein expression of GATA4 was found in normal lung tissues, while medium protein expression was exhibited in LC tissues. However, the protein expression of GATA5 in LC is not described.

### Relationship between the GATAs mRNA expression levels and the clinicopathological parameters of patients with LC

**Figure 4:**
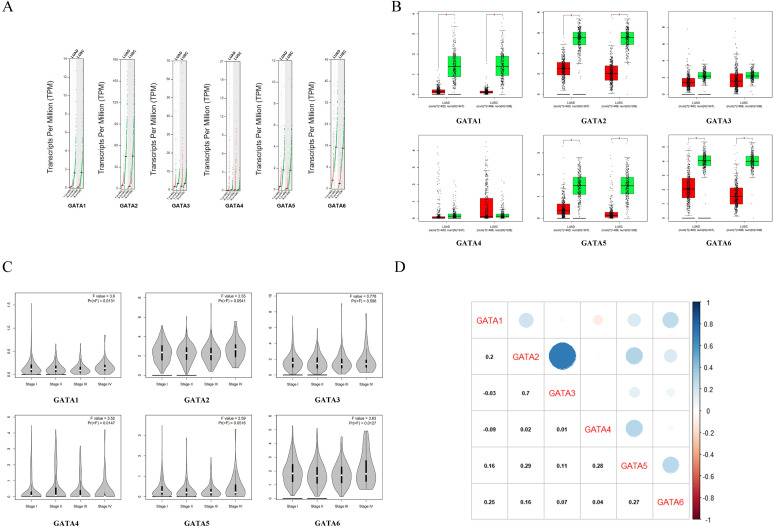
** The mRNA differential expression levels of GATAs in accordance with different cancer types or pathological stages and the correlation analysis between GATAs (Gene Expression Profiling Interactive Analysis, GEPIA). (A-B)** The expression of GATAs in LC. LUAD, Lung adenocarcinoma. LUSC, Lung squamous cell carcinoma.*P < 0.05. **(C)** Correlation between GATAs expression and tumor stage in LC patients.*P < 0.05. **(D)** The correlations of GATAs with each other by analyzing their mRNA expression.

We initially compared the mRNA expression of GATAs in LC and normal lung tissue again by using GEPIA dataset. The results implied that GATA1, GATA2, GATA5 and GATA6 were remarkably decreased in lung adenocarcinoma and squamous cell lung carcinoma tissues in comparison with normal lung tissues, whereas there was no significant difference in the expressions of GATA3 and GATA4 between LC and normal lung tissue (**Figure 4A-B**). Next, the relationship between the expression of GATAs and tumor stages in lung adenocarcinoma and squamous cell lung carcinoma was analyzed. The results indicated that the expression of GATA1, GATA4 and GATA6 remarkably varied across the tumor stages, whereas GATA2, GATA3 and GATA5 demonstrated no significantly changes in different tumor stages (**Figure 4C**). Moreover, the Pearson correlation coefficients among GATAs were calculated by using “correlation analysis” in GEPIA. The results indicated that GATA1 may be negatively correlated with GATA3 and GATA4 with coefficients of -0.03 and -0.09, respectively (**Figure 4D**). And other coefficients ranged from 0.01 (GATA3 vs. GATA4) to 0.7 (GATA2 vs. GATA3) (**Figure 4D**).

### Prognostic value of GATAs in patients with LC

**Figure 5:**
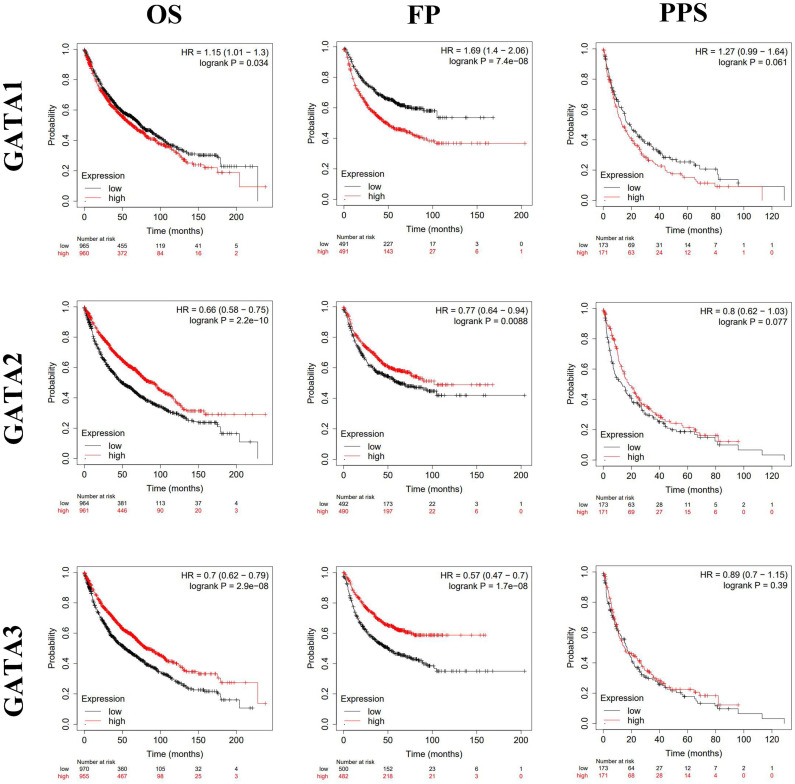
The prognostic value of mRNA level of GATA1-3 in patients with LC (Kaplan-Meier plotter). *P < 0.05.

**Figure 6:**
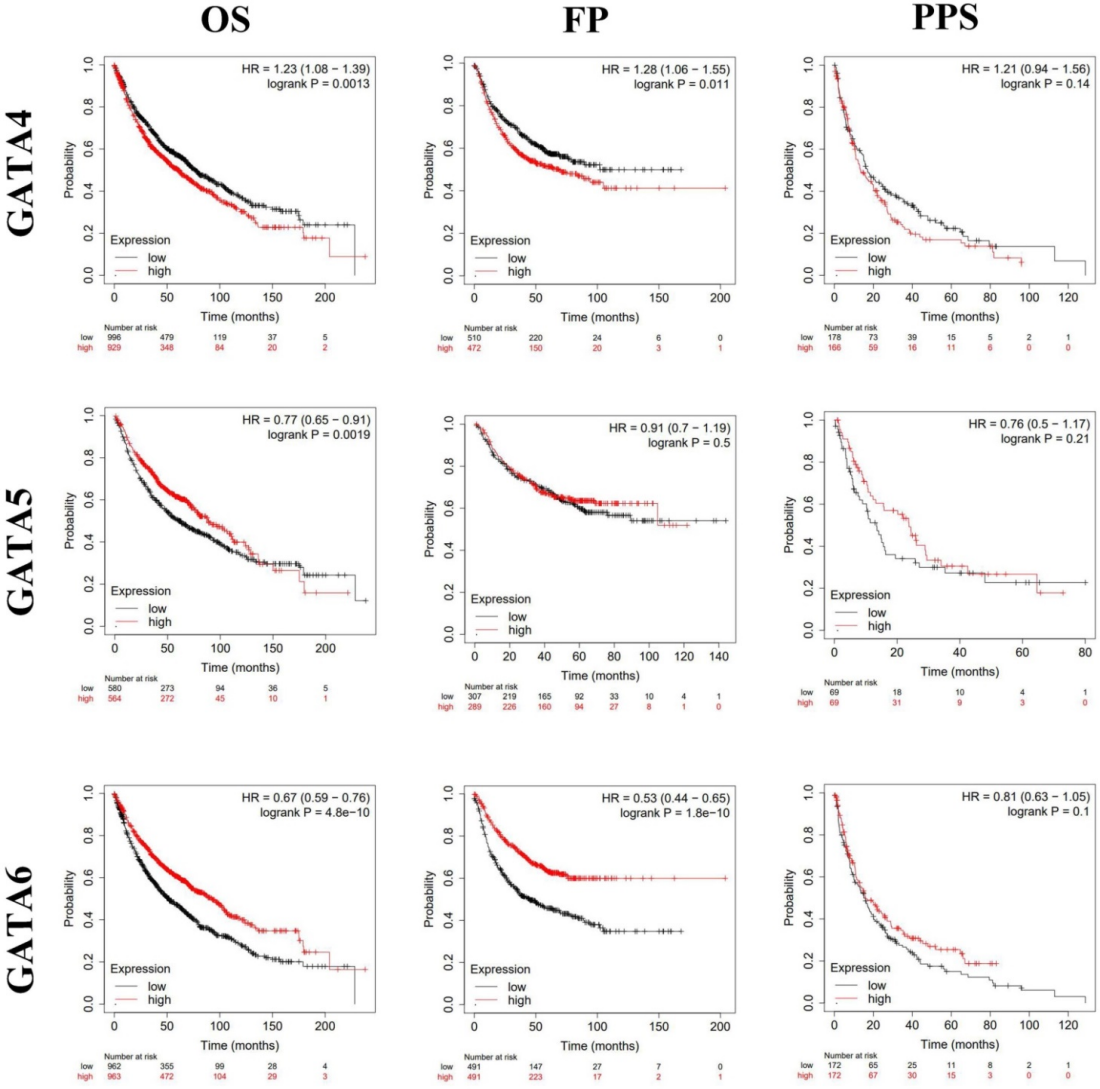
The prognostic value of mRNA level of GATA4-6 in patients with LC (Kaplan-Meier plotter). *P < 0.05.

By using Kaplan-Meier plotter analysis, we explored the prognostic value of GATAs in all patients with LC. The results demonstrated that the increased GATA2/3/6 mRNA levels and the decreased GATA1/4 mRNA levels were notably relevant to the OS and FP of all of the patients with LC, whereas the increased GATA5 mRNA levels were significantly associated with the OS of all of the patients with LC. However, there was no correlation between the mRNA expression of individual GATA and the PPS of LC patients (**Figure 5-6**). To further determine prognostic value of GATAs in patients with LC, we obtained similar results from some microarray datasets of GEO database (**Supplementary Figure 1A-B**). The results showed that the increased GATA3/5 mRNA levels were relevant to better survival probability in lung adenocarcinoma. And in squamous cell lung carcinoma, the increased GATA5/6 also indicated better survival probability (**Supplementary Figure 1C-D**). On the contrary, the increased GATA1 was associated with poor survival probability, whereas there was no significant relationship between GATA2 expression and survival probability (**Supplementary Figure 1E-F**). In addition, we also analyzed the prognostic values of GATAs in different subtypes of LC, that is, different histology, clinical stages, pathological grades, and smoking history, which can be obtained in Kaplan-Meier plotter. As shown in **Table 2**, the increased GATA2/3/5/6 mRNA levels and the decreased GATA4 mRNA levels in lung adenocarcinoma were remarkably related to improved OS. Furthermore, the decreased mRNA expression of GATA1 and the increased mRNA expression of GATA2 in lung adenocarcinoma patients, and the decreased levels of GATA5 and the increased levels of GATA6 in both lung adenocarcinoma and squamous cell lung carcinoma patients were significantly related to longer FP. As shown in **Table 3**, elevated mRNA levels of GATA2/3/5/6 and low levels of GATA4 were connected with better OS in stage 1, while high level of GATA6 indicated better OS in stage 2 and high level of GATA2 predicted better OS in stage 4. With respect to pathological grades, high level of GATA1 was associated with poor OS in grade I. Additionally, elevated expression of GATA2/3/6 and low levels of GATA1 were correlated to better OS in both those smoked and never smoked, while increased levels of GATA4 were related to poor OS in those smoked. Regarding FP (**Table 4**), elevated mRNA levels of GATA6 was associated with longer FP in stage 1, while elevated mRNA levels of GATA1 was linked to shorter PFS in grade 1. Moreover, elevated expression of GATA1 and low levels of GATA6 predicted shorter PFS in both those smoked and never smoked. Then, high mRNA expression of GATA3 and low levels of GATA4 were related to longer FP in those smoked, whereas increased expression of GATA2 predicted better FP in those never smoked. With reference to PPS (**Table 5**), high level of GATA1/2/5 and low expression of GATA4 were correlated to better PPS in stage 1, while elevated mRNA levels of GATA3 predicted better PPS in those never smoked. To sum up, these results emphasized the prognostic value of the mRNA expression levels of GATAs on predicting survival of LC patient, implying that GATAs may be potential biomarkers.

### Genetic alteration analysis of GATAs in patients with LC

**Figure 7:**
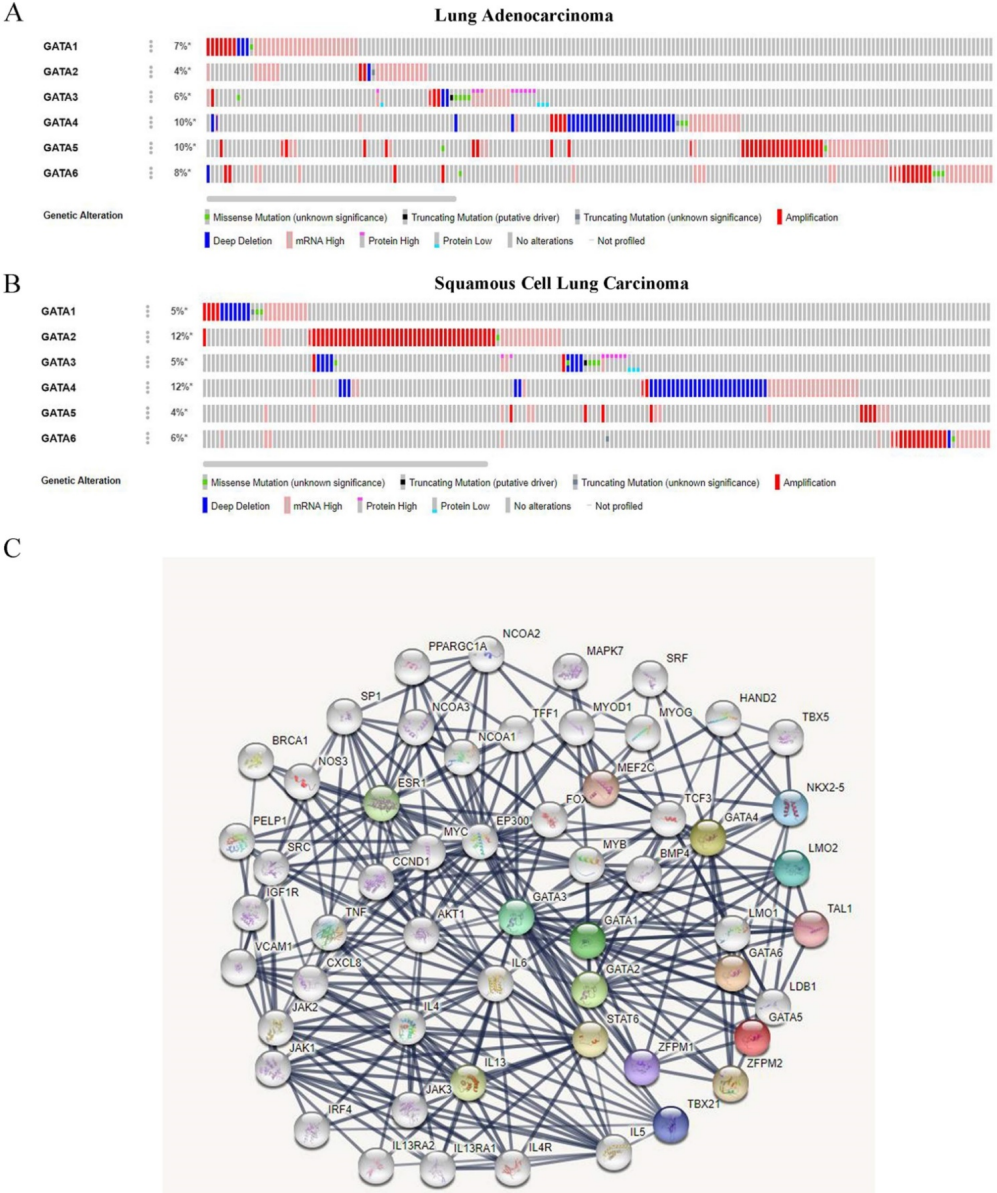
**The genetic alteration and protein-protein interaction (PPI) of GATAs. (A)** The genetic alteration analysis of GATAs in patients with LC (lung adenocarcinoma and squamous cell lung carcinoma). **(B)** The network of 6 GATA members and 50 proteins that significantly interacted with GATAs (String).

We analyzed the genetic alterations of GATAs in LC patients using the cBioPortal online tool. GATAs were analyzed in 586 samples of 584 patients from the TCGA databases of lung adenocarcinoma (TCGA, Firehose Legacy) and 511 samples of 511 patients from squamous cell lung carcinoma (TCGA, Firehose Legacy), and the alteration rates were 30.99% (181/584) and 35.23% (180/511), respectively. The mutation rates of GATAs in lung adenocarcinoma and squamous cell lung carcinoma were inconsistent, and the top 3 are listed respectively. Among them, the alteration rates of GATA4/5/6 were 10, 10, and 8%, respectively in lung adenocarcinoma (**Figure 7A**). Furthermore, the alteration rates of GATA2/4/6 were 12, 12, and 6%, respectively in squamous cell lung carcinoma (**Figure 7B**).

### GO enrichment and KEGG pathway analysis of Protein-Protein interaction of GATAs

A PPI network consisting of 6 GATAs and 50 proteins that distinctly interacted with GATAs was constructed using the String database [PPI enrichment *P<*1.0e-16]. The top ten of the network graphic showed that LIM domain only 2 (LMO2), NK2 homeobox 5 (NKX2-5), T-box transcription factor 21 (TBX21), zinc finger protein, FOG family member 1/2 (ZFPM1/2), T-cell acute lymphocytic leukemia protein 1 (TAL1), Myocyte-specific enhancer factor 2C (MEF2C), Signal transducer and activator of transcription 6 (STAT6), Interleukin-13 (IL13) and estrogen receptor 1 (ESR1) with GATAs (**Figure 7C**).

**Figure 8:**
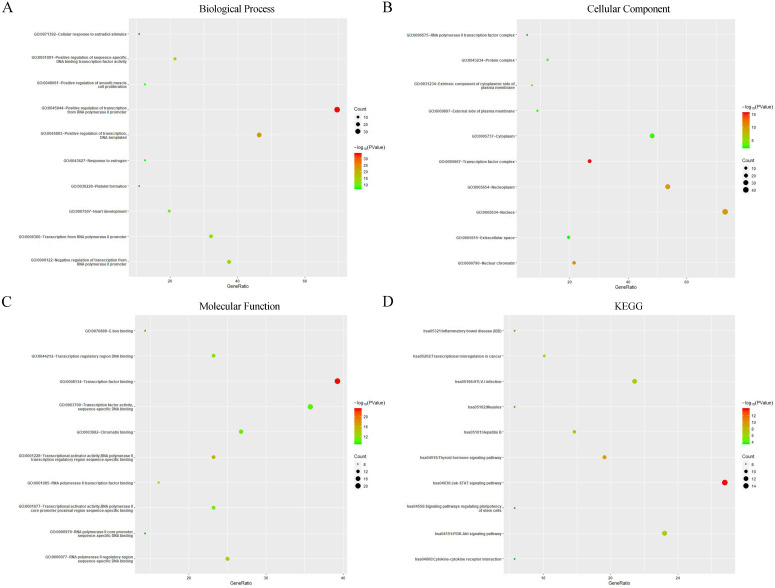
** Gene Ontology (GO) enrichment and Kyoto Encyclopedia of Genes and Genomes (KEGG) pathway analysis of GATAs and their interactors (Database for Annotation, Visualization, and Integrated Discovery, DAVID).** GO enrichment analysis of target genes based on following three aspects: **(A)** Biological Process, **(B)** Cellular Component, and **(C)** Molecular Function. **(D)** KEGG pathway enrichment analysis of target genes.

Next, GO enrichment and KEGG pathway analysis of GATAs and their interactors were achieved using Database for Annotation, Visualization, and Integrated Discovery (DAVID). GO enrichment analysis predicted the functional roles of target host genes on account of three orientations, including biological processes (BP), cellular components (CC) and molecular functions (MF). The results showed that the three major biological processes of target genes were positive regulation of transcription from RNA polymerase II promoter, positive regulation of transcription, DNA-templated and positive regulation of sequence-specific DNA binding transcription factor activity (**Figure 8A**). Cellular components such as transcription factor complex, nuclear chromatin and nucleus were remarkably regulated by GATAs and their interacting neighbors (**Figure 8B**), while transcription factor binding, transcriptional activator activity, RNA polymerase II transcription regulatory region sequence-specific binding and RNA polymerase II regulatory region sequence-specific DNA binding were their primary molecular functions (**Figure 8C**). Furthermore, KEGG pathway analysis indicated that Jak-STAT signaling pathway, Thyroid hormone signaling pathway and Inflammatory bowel disease were major pathways associated with GATAs and their interacting neighbors (**Figure 8D**).

## Discussion

The dysregulation of GATAs has been found in multiple cancers. Although the function of GATAs in the progress of several cancers has been partially identified, the distinct roles of six GATAs in the tumorigenesis and prognosis of LC are yet to be elucidated. In the present study, it is the first time to probe into the diverse expression patterns, prognostic values (OS, FP and PPS), genetic alterations and PPI networks of GATAs in LC patients through several open online databases.

GATA1, the first recognized member of the GATA family, functions as both tumor suppressor and promotor and has been largely reported to have important roles in the development, progression and prognosis of cancers 33-36. However, its expression pattern and prognostic value in LC have been rarely reported. Only one study by Wang 37 reported that suppression of endogenous GATA-1 gene expression was found in lung adenocarcinoma, which might be connected with the upregulation of IRF-3. Similarly, in the current study, CCLE datasets and GEPIA datasets indicated that the expression of GATA1 was lower in LC tissues and cell lines than in normal controls. Moreover, an increased level of GATA1 was correlated to unfavorable LS and PFS in LC, especially the PFS in adenocarcinoma subtypes, and tumor stages.

GATA2 was reported to function early in hematopoiesis 38 and was lowly expressed in several tumors, such as hepatocellular carcinoma 39, gastric cancer 40, clear cell renal cell carcinoma 35, and highly expressed in prostate cancer 41. GATA2 was significantly downregulated in both human and mouse lung tumors and its further suppression was not an effective treatment for KRAS mutant lung cancer 12. Conversely, Kumar et al. 13 found that GATA2 was essential for survival of KRAS mutant NSCLC and the inhibition of GATA2 regulated pathways can play an inhibitory effect on tumor, while nanoparticles carrying siGATA2 lent significant therapeutic promise in KRAS mutant NSCLC therapy. In our report, ONCOMINE datasets, Human Protein Atlas datasets and GEPIA datasets reveled that the expression of GATA2 was lower in human LC compared with in normal tissues. However, CCLE datasets indicated that GATA2 was higher in LC cell lines. Although it is not explicit, we ascribed the inconsistent discoveries to the different cell lines or background heterogeneity between different databases. Moreover, increased levels of GATA2 were associated with favorable LS and PFS in LC, especially in adenocarcinoma subtypes. In addition, our results showed that the genetic alteration rate of GATA2 was 12% and the amplification accounted for most changes.

GATA3 is a unique member of the GATA family that is largely reported to play important roles in the pathogenesis of cancer, including tumorigenesis, tumor differentiation, EMT, and metastasis through regulation of miscellaneous target genes, especially in breast cancer 6, 42, 43. Nonetheless, the cognition of the unique role of GATA3 in LC is still in its infancy. In our study, GATA3 expression levels showed a contradictory result in LC compared with normal tissues based on ONCOMINE datasets, while Human Protein Atlas datasets and GEPIA datasets showed no statistical difference of GATA3 expression between LC and normal tissues. Based on the discovery that GATA3 was largely expressed in distant metastasis but not in lung tumors, the study believed that GATA3 was necessary for EMT, invasion and metastasis of lung adenocarcinoma cells 44, while Lysyl hydroxylases including LH2 and LH3 played a distinct role as the direct transcription target of GATA3 to drive lung cancer cell metastasis 45. Furthermore, GATA3 acetylation mediated by acetyltransferase CBP on lysine 119 could inhibit the migration and invasion of lung adenocarcinoma cells 46. These findings implied that GATA3 may be a new prognostic marker for LC patients. Likewise, our results showing that a high GATA3 expression was remarkably connected with better OS and FP.

GATA4 is considered necessary for normal pulmonary lobar development 47 and is abnormally expressed and involved in cancer-associated cellular processes in numerous malignancies 48-52. Although the methylation of GATA4 seems to be a key part in the development of LC, including DNA damage and metastasis 53-55, further studies are needed to investigate the expression of GATA4 and its prognostic value in LC. GATA4 was identified as an essential tumor suppressor and its expression was uniformly decreased in human LC cell lines compared to normal lung epithelial cell lines 16, which was consistent with the results of our study based on CCLE datasets. Meanwhile, Gao et al. 16 also suggested that decreased GATA4 level in clinical specimens predicted poor prognosis. On the contrary, our results demonstrated that low expression of GATA4 was relevant to better OS and FP, especially OS in stage 1. In addition, the mutation rates of GATA4 in lung adenocarcinoma and squamous cell lung carcinoma were 10% and 12%, respectively, which were both the highest mutation rates compared with other GATAs and the deep deletion accounted for most changes. The high mutation rate of GATA4 in LC may account for the discrepancy in the above results.

GATA5 is located at chromosome 20q13, a locus which is often amplified and methylated in multiple cancer types, including LC 56-58. In 63 cases of primary lung cancers, GATA-5 promoter methylation was detected in (26 of 63) 41% 59. Compared with tumor tissue, the assessment of the methylation status of GATA5 combined with the other three genes p16, DAPK, PAX5β in sputum can be considered as more effective for advanced lung cancer tissue where biopsy is not feasible 60. Consistently, in this study, the genetic alteration rate of GATA5 was 10% in lung adenocarcinoma and the amplification accounted for most changes. Furthermore, the decreased mRNA expression of GATA5 was found in LC issues and cell lines and associated with poor OS, especially in stage 1.

GATA6 is involved in cell lineage differentiation and organ formation in numerous tissue types and had a contradictory effect on tumor development in accordance with tumor origin. GATA6 was remarkably reduced in squamous cell lung carcinoma tissues and can inhibit the proliferation and migration of squamous cell lung carcinoma cells by transcriptionally restraining the expression of Shh, which imply that targeting GATA6 may be a potential therapy approach for squamous cell lung carcinoma 11. Similarly, our study indicated that the mRNA and protein levels of GATA6 in different subtypes of LC were prominently lower compared with those in normal samples, while there was no significant change in the LC cell lines. In addition, only 47 of 348 cases of lung adenocarcinoma had positive GATA6 expression (13.5%) and GATA6 has no significant influence on OS or DFS 17. Conversely, our report suggested that GATA6 had obvious prognostic value for LC and high expression GATA6 predicated better OS and FP, especially in stag1. Moreover, detection of GATA6 in exhaled breath condensate seem to be an efficient diagnosis for non-invasive LC 61, and GATA6 can induce terminal differentiation and growth arrest in Tyrosine Kinase Inhibitors (TKI) resistant NSCLC cells by inhibiting EGFR and Wnt signaling activation 62.

Despite the abundance of our findings, there are still some limitations in the present study. First, due to the inevitable background heterogeneity between different databases, there may be some inconsistencies in our results. Second, the potential diagnostic and therapeutic roles of GATAs in LC were not assessed. Finally, the potential mechanisms of distinct GATAs in LC were not explored in our study. To address these issues, we prepare to carry out elaborate studies to further validate and explore potential roles and mechanisms of GATAs in LC in the near future.

In conclusion, we in detail analyzed the differential expression patterns, prognostic value, genetic alterations and PPI networks of GATAs in LC and provided a comprehensive cognition of the complex and heterogeneous molecular biological properties of LC. This integrated bioinformatic analysis demonstrated that GATA1-4, and 6 may be new prognostic biomarkers, and GATA2/5/6 may be potential targets for personalized therapy for patients with LC. However, further studies are requisite to analyze the mechanism of their carcinogenicity and investigate novel drug treatment. Finally, these findings would conduce to a better understanding of the unique roles of GATAs in LC.

## Supplementary Material

Supplementary figures and tables.

## Figures and Tables

**Table 1:** Significant changes of GATAs expression in transcription level between different types of lung cancer and lung tissues (ONCOMINE database)

Types of lung cancer vs. lung	Fold change	*P* value	T-test	Ref.
**GATA1**				
NA	NA	NA	NA	NA
**GATA2**				
Small Cell Lung Carcinoma vs. Normal	-13.297	3.86E-8	-8.225	Bhattacharjee 21
Squamous Cell Lung Carcinoma vs. Normal	-8.029	4.70E-6	-5.175	Bhattacharjee 21
Lung Adenocarcinoma vs. Normal	-6.027	5.83E-6	-5.966	Bhattacharjee 21
Lung Carcinoid Tumor vs. Normal	-12.984	2.37E-8	-7.415	Bhattacharjee 21
Lung Adenocarcinoma vs. Normal	-4.430	4.55E-12	-10.759	Stearman 22
Lung Adenocarcinoma vs. Normal	-2.451	8.09E-12	-8.575	Su 23
Lung Adenocarcinoma vs. Normal	-2.552	9.10E-22	-13.626	Landi 24
Lung Adenocarcinoma vs. Normal	-2.361	3.99E-7	-6.070	Beer 25
Lung Adenocarcinoma vs. Normal	-2.218	5.40E-21	-12.451	Selamat 22
Squamous Cell Lung Carcinoma vs. Normal	-3.780	3.86E-18	-13.540	Hou 28
Lung Adenocarcinoma vs. Normal	-2.475	9.70E-15	-9.386	Hou 28
Large Cell Lung Carcinoma vs. Normal	-4.077	1.49E-11	-11.069	Hou 28
Squamous Cell Lung Carcinoma vs. Normal	-3.551	1.36E-4	-5.206	Garber 29
Large Cell Lung Carcinoma vs. Normal	-2.194	0.005	-3.400	Garber 29
Lung Adenocarcinoma vs. Normal	-2.318	0.002	-3.998	Garber 29
Lung Adenocarcinoma vs. Normal	-3.332	2.69E-13	-13.181	Okayama 26
Squamous Cell Lung Carcinoma vs. Normal	-2.142	0.001	-6.351	Wachi 27
**GATA3**				
Large Cell Lung Carcinoma vs. Normal	2.590	0.006	3.583	Garber 29
Lung Adenocarcinoma vs. Normal	-2.996	2.02E-6	-5.186	Beer 25
**GATA4**				
NA	NA	NA	NA	NA
**GATA5**				
NA	NA	NA	NA	NA
**GATA6**				
Lung Adenocarcinoma vs. Normal	-5.083	1.08E-13	-8.997	Beer 25
Squamous Cell Lung Carcinoma vs. Normal	-4.710	2.38E-5	-12.129	Wachi 27
Lung Adenocarcinoma vs. Normal	-3.568	1.76E-25	-14.854	Landi 24
Squamous Cell Lung Carcinoma vs. Normal	-3.793	3.84E-6	-6.424	Garber 29
Large Cell Lung Carcinoma vs. Normal	-3.039	9.16E-4	-5.191	Garber 29
Lung Adenocarcinoma vs. Normal	-3.244	1.10E-5	-7.718	Garber 29
Small Cell Lung Carcinoma vs. Normal	-5.177	0.002	-5.639	Garber 29
Lung Adenocarcinoma vs. Normal	-2.368	1.01E-16	-11.238	Okayama 26
Large Cell Lung Carcinoma vs. Normal	-8.949	7.04E-12	-13.317	Hou 28
Lung Adenocarcinoma vs. Normal	-4.042	1.39E-12	-9.103	Hou 28
Squamous Cell Lung Carcinoma vs. Normal	-6.186	5.34E-13	-11.662	Hou 28
Squamous Cell Lung Carcinoma vs. Normal	-2.600	0.004	-2.779	Bhattacharjee 21
Lung Adenocarcinoma vs. Normal	-3.392	1.17E-6	-5.735	Su 23

**Table 2:** The prognostic values of GATAs in different pathological subtypes lung cancer (Kaplan-Meier plotter)

GATAs	Histology	OS			FP			PPS		
		Cases	HR (95% CI)	*P* value	Cases	HR (95% CI)	*P* value	Cases	HR (95% CI)	*P* value
GATA1	Adenocarcinoma	719	1.11 (0.88-1.4)	0.39	461	1.51 (1.1-2.07)	0.0095	125	0.83 (0.52-1.33)	0.43
	Squamous cell carcinoma	524	0.97 (0.77-1.23)	0.82	141	0.89 (0.53-1.49)	0.66	20	1.56 (0.55-4.42)	0.40
GATA2	Adenocarcinoma	719	0.71 (0.56-0.9)	0.004	461	0.51 (0.37-0.71)	3.6e-5	125	0.75 (0.47-1.2)	0.23
	Squamous cell carcinoma	524	0.86 (0.68-1.09)	0.21	141	1.09 (0.65-1.82)	0.75	20	1.38 (0.48-3.94)	0.54
GATA3	Adenocarcinoma	719	0.6 (0.47-0.76)	2.4e-5	461	0.82 (0.6-1.12)	0.21	125	0.73 (0.46-1.16)	0.18
	Squamous cell carcinoma	524	0.91 (0.72-1.15)	0.42	141	0.69 (0.41-1.16)	0.16	20	1.1 (0.37-3.33)	0.86
GATA4	Adenocarcinoma	719	1.34 (1.06-1.69)	0.014	461	1.27 (0.93-1.74)	0.13	125	1.35 (0.84-2.18)	0.22
	Squamous cell carcinoma	524	0.96 (0.76-1.21)	0.71	141	0.84 (0.51-1.41)	0.51	20	2.01 (0.68-5.93)	0.2
GATA5	Adenocarcinoma	719	0.59 (0.46-0.76)	3.3e-5	461	0.69 (0.5-0.95)	0.024	125	0.81 (0.5-1.32)	0.39
	Squamous cell carcinoma	524	1.14 (0.83-1.55)	0.42	141	1.78 (1.06-3.01)	0.027	20	1.18 (0.42-3.28)	0.76
GATA6	Adenocarcinoma	719	0.53 (0.42-0.68)	1.5e-7	461	0.62 (0.45-0.85)	0.0028	125	0.79 (0.5-1.26)	0.32
	Squamous cell carcinoma	524	1.05 (0.83-1.33)	0.68	141	0.55 (0.32-0.92)	0.022	20	2.05 (0.68-6.16)	0.19

**Table 3:** The relationship between GATAs and OS in other different subtypes of lung cancer (Kaplan-Meier plotter)

Subtypes	Cases	GATA1		GATA2		GATA3		GATA4		GATA5		GATA6	
		HR (95% CI)	*P*	HR (95% CI)	*P*	HR (95% CI)	*P*	HR (95% CI)	*P*	HR (95% CI)	*P*	HR (95% CI)	*P*
**Stage**													
1	577	1.12 (0.86-1.47)	0.41	0.48 (0.36-0.64)	2.3e-7	0.5 (0.37-0.66)	5.4e-7	1.48 (1.13-1.94)	0.0043	0.42 (0.3-0.59)	2.0e-7	0.39 (0.3-0.52)	2.1e-11
2	244	1.4 (0.97-2.02)	0.07	0.77 (0.53-1.11)	0.16	0.86 (0.6-1.24)	0.41	1.17 (0.81-1.68)	0.41	0.81 (0.51-1.28)	0.36	0.63 (0.43-0.91)	0.012
3	70	0.81 (0.47-1.41)	0.46	0.58 (0.33-1)	0.048	0.78 (0.45-1.35)	0.38	0.81 (0.47-1.4)	0.45	0.72 (0.36-1.45)	0.36	0.99 (0.58-1.7)	0.98
4	4	NA	NA	NA	NA	NA	NA	NA	NA	NA	NA	NA	NA
**Grade**													
I	201	1.9 (1.31-2.76)	5.6e-4	0.8 (0.56-1.15)	0.22	0.98 (0.69-1.4)	0.92	0.98 (0.68-1.4)	0.91	NA	NA	1.01 (0.7-1.44)	0.98
II	310	0.94 (0.69-1.29)	0.71	0.74 (0.54-1.01)	0.061	0.83 (0.61-1.14)	0.25	0.82 (0.6-1.13)	0.22	NA	NA	0.97 (0.71-1.32)	0.84
III	77	1.2 (0.62-2.32)	0.58	0.52 (0.27-1.02)	0.053	0.53 (0.27-1.03)	0.056	1.51 (0.78-2.92)	0.22	NA	NA	0.61 (0.32-1.19)	0.14
**Smoking history**													
Exclude those never smoked	820	1.43 (1.16-1.76)	6.9e-4	0.66 (0.53-0.81)	7.9e-5	0.75 (0.61-0.93)	0.0071	1.29 (1.05-1.59)	0.014	0.72 (0.47-1.08)	0.11	0.71 (0.57-0.87)	0.0011
Only those never smoked	205	2.46 (1.37-4.42)	0.0019	0.39 (0.21-0.72)	0.0017	0.34 (0.19-0.63)	0.0003	1.22 (0.7-2.12)	0.49	0.44 (0.17-1.12)	0.077	0.41 (0.22-0.74)	0.0023

**Table 4:** The relationship between GATAs and FP in other different subtypes of lung cancer (Kaplan-Meier plotter)

Subtypes	Cases	GATA1		GATA2		GATA3		GATA4		GATA5		GATA6	
		HR (95% CI)	*P*	HR (95% CI)	*P*	HR (95% CI)	*P*	HR (95% CI)	*P*	HR (95% CI)	*P*	HR (95% CI)	*P*
**Stage**													
1	325	1.26 (0.81-1.95)	0.3	0.67 (0.43-1.05)	0.076	1.01 (0.65-1.56)	0.98	1.18 (0.76-1.83)	0.46	0.79 (0.51-1.23)	0.29	0.63 (0.41-0.98)	0.04
2	130	1.39 (0.83-2.34)	0.2	0.69 (0.41-1.17)	0.16	1.23 (0.74-2.07)	0.42	1.09 (0.65-1.83)	0.74	1.02 (0.59-1.73)	0.96	0.9 (0.53-1.5)	0.68
3	19	NA	NA	NA	NA	NA	NA	NA	NA	NA	NA	NA	NA
4	0	NA	NA	NA	NA	NA	NA	NA	NA	NA	NA	NA	NA
**Grade**													
I	140	1.61 (1.04-2.51)	0.033	1.05 (0.68-1.63)	0.82	1.05 (0.68-1.62)	0.83	0.97 (0.63-1.5)	0.88	NA	NA	0.93 (0.6-1.44)	0.76
II	165	0.74 (0.49-1.13)	0.16	0.7 (0.46-1.06)	0.089	0.74 (0.49-1.13)	0.16	1.15 (0.76-1.74)	0.51	NA	NA	0.67 (0.44-1.02)	0.061
III	51	1.16 (0.52-2.59)	0.71	1.5 (0.66-3.4)	0.32	1.06 (0.47-2.36)	0.89	1.77 (0.78-4.05)	0.17	NA	NA	0.95 (0.43-2.12)	0.9
**Smoking history**													
Exclude those never smoked	603	1.91 (1.49-2.44)	2.4e-7	0.82 (0.64-1.04)	0.11	0.62 (0.48-0.79)	1.1e-4	1.45 (1.14-1.85)	0.0027	1.09 (0.74-1.61)	0.67	0.62 (0.48-0.79)	0.0001
Only those never smoked	193	1.8 (1.11-2.92)	0.015	0.48 (0.29-0.79)	0.003	0.63 (0.39-1.01)	0.053	0.87 (0.54-1.41)	0.58	0.59 (0.31-1.13)	0.11	0.6 (0.37-0.97)	0.037

**Table 5:** The relationship between GATAs and PPS in other different subtypes of lung cancer (Kaplan-Meier plotter)

Subtypes	Cases	GATA1		GATA2		GATA3		GATA4		GATA5		GATA6	
		HR (95% CI)	*P*	HR (95% CI)	*P*	HR (95% CI)	*P*	HR (95% CI)	*P*	HR (95% CI)	*P*	HR (95% CI)	*P*
**Stage**													
1	78	0.54 (0.29-0.99)	0.043	0.44 (0.23-0.81)	0.0074	0.69 (0.38-1.26)	0.23	1.96 (1.05-3.66)	0.032	0.37 (0.2-0.71)	0.0016	0.74 (0.41-1.35)	0.32
2	58	1.46 (0.76-2.8)	0.25	0.7 (0.37-1.34)	0.28	0.54 (0.27-1.05)	0.065	1.09 (0.57-2.1)	0.79	1.59 (0.81-3.15)	0.18	0.7 (0.36-1.34)	0.28
3	10	NA	NA	NA	NA	NA	NA	NA	NA	NA	NA	NA	NA
4	0	NA	NA	NA	NA	NA	NA	NA	NA	NA	NA	NA	NA
**Grade**													
I	79	0.94 (0.58-1.54)	0.81	0.73 (0.45-1.19)	0.2	1.13 (0.69-1.85)	0.62	1.18 (0.73-1.93)	0.5	NA	NA	1.14 (0.7-1.86)	0.6
II	89	1.23 (0.76-2)	0.4	0.97 (0.6-1.57)	0.91	0.74 (0.46-1.19)	0.21	0.99 (0.62-1.6)	0.98	NA	NA	1.18 (0.73-1.9)	0.5
III	24	1.34 (0.49-3.62)	0.57	0.38 (0.13-1.1)	0.065	0.65 (0.24-1.78)	0.4	0.67 (0.25-1.81)	0.43	NA	NA	0.94 (0.33-2.65)	0.9
**Smoking history**													
Exclude those never smoked	254	1.16 (0.87-1.55)	0.3	0.83 (0.62-1.11)	0.21	1.08 (0.81-1.44)	0.61	1.17 (0.88-1.57)	0.28	1.01 (0.61-1.68)	0.96	1.01 (0.76-1.35)	0.93
Only those never smoked	67	1.68 (0.9-3.14)	0.1	0.77 (0.41-1.45)	0.42	0.45 (0.24-0.85)	0.012	1.54 (0.82-2.9)	0.18	0.58 (0.23-1.43)	0.23	0.64 (0.34-1.21)	0.17
