# Supplementary material for: Comprehensive Analysis of Expression and Prognostic Value of GATAs in Lung Cancer
Source: J Cancer. 2021 May 5;12(13):3862–76. doi: 10.7150/jca.52623 (PMC8176258; doi:10.7150/jca.52623)
Supplement: Supplementary file 1 — Supplementary figures and tables. [file jcav12p3862s1.pdf]

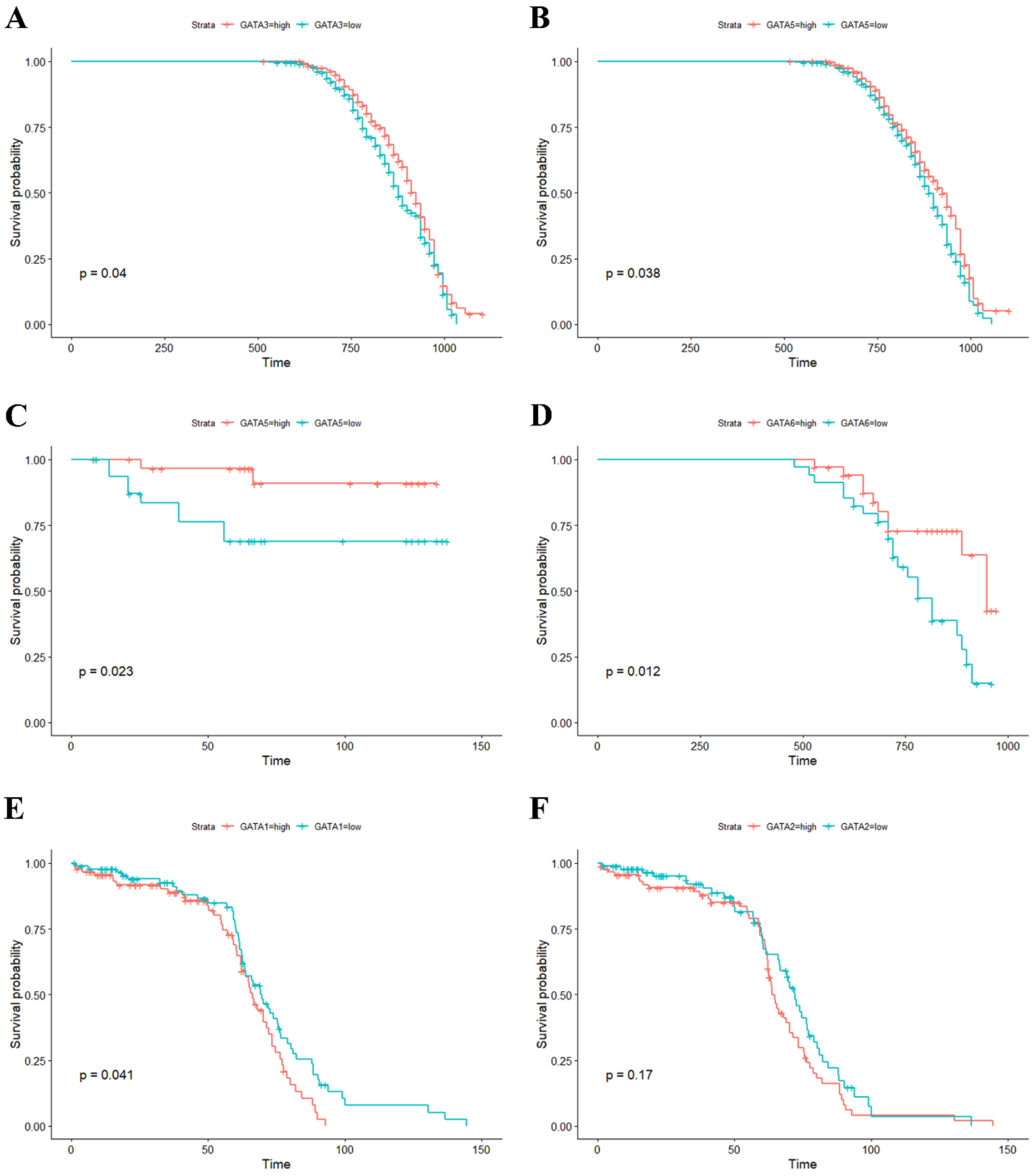

Supplementary Figure 1. The prognostic value of mRNA level of GATA1-6 in patients with LC (The Gene Expression Omnibus (GEO) database). *$P < 0.05$.
